# Mineralocorticoid Receptor Antagonists, Blood Pressure, and Outcomes in Heart Failure With Reduced Ejection Fraction

**DOI:** 10.1016/j.jchf.2019.09.011

**Published:** 2020-03

**Authors:** Matteo Serenelli, Alice Jackson, Pooja Dewan, Pardeep S. Jhund, Mark C. Petrie, Patrick Rossignol, Gianluca Campo, Bertram Pitt, Faiez Zannad, João Pedro Ferreira, John J.V. McMurray

**Affiliations:** a British Heart Foundation Cardiovascular Research Centre, University of Glasgow, Glasgow, United Kingdom; b Cardiovascular Centre of Ferrara University, Ferrara University, Ferrara, Italy; c Centre d'Investigations Cliniques Plurithématique 1433, Université de Lorraine INSERM, CHRU de Nancy, F-CRIN INI-CRCT, Nancy France; d Gruppo Villa Maria Care & Research, Maria Cecilia Hospital, Cotignola (RA), Italy; e Department of Internal Medicine-Cardiology, University of Michigan School of Medicine, Ann Arbor, Michigan

**Keywords:** aldosterone, blood pressure, ejection fraction, eplerenone, heart failure, mineralocorticoid receptor, spironolactone, ACE, angiotensin converting-enzyme, ARBs, angiotensin receptor blockers, BP, blood pressure, eGFR, estimated glomerular filtration rate, HFrEF, heart failure with reduced ejection fraction, LVEF, left ventricular ejection fraction, MRAs, mineralocorticoid receptor antagonists, NYHA, New York heart association, SBP, systolic blood pressure

## Abstract

**Objectives:**

The purpose of this study was to investigate the effects of mineralocorticoid receptor antagonists (MRAs) on systolic blood pressure (SBP) and outcomes according to baseline SBP in patients with heart failure with reduced ejection fraction (HFrEF).

**Background:**

MRAs are greatly underused in patients with HFrEF, often because of fear of adverse events. Concern about hypotension has been raised by the demonstration that MRAs are particularly effective treatment for resistant hypertension.

**Methods:**

The effect of MRA therapy was studied in 4,396 patients with HFrEF randomized in the RALES (Randomized Aldactone Evaluation Study) and EMPHASIS-HF (Eplerenone in Mild Patients Hospitalization and Survival Study in Heart Failure) trials.

**Results:**

Mean SBP change from baseline to 6 months was +1.4 ± 18.1 mm Hg in the placebo group and −1.2 ± 17.9 mm Hg in the MRA group. The between-treatment difference was 2.6 mm Hg (95% confidence interval [CI]: 1.5 to 3.6; p < 0.001). All outcomes were reduced by MRA therapy overall, with consistent effects across SBP categories (e.g., all-cause mortality, overall hazard ratio [HR] of 0.72; 95% CI: 0.64 to 0.82; p < 0.001; SBP ≤105 mm Hg; HR: 0.72; 95% CI: 0.56 to 0.94; SBP >105 to ≤115 mm Hg; HR: 0.78; 95% CI: 0.60 to 1.02; SBP >115 to ≤125 mm Hg; HR: 0.71; 95% CI: 0.53 to 0.94; SBP >125 to ≤135 mm Hg; HR: 0.79; 95% CI: 0.57 to 1.10; and SBP > 135 mm Hg; HR: 0.67; 95% CI: 0.50 to 0.90; p for interaction = 0.95). Hypotension was infrequent and not more common with MRA therapy than with placebo, overall (4.6% vs. 3.9%; p = 0.25) or in any SBP category.

**Conclusions:**

MRA treatment had little effect on SBP in patients with HFrEF, and the clinical benefits were not modified by baseline SBP. MRA treatment infrequently caused hypotension, even when the baseline SBP was low. The treatment discontinuation rates between MRA and placebo therapy were similar. Low SBP is not a reason to withhold MRA therapy in patients with HFrEF.

Although mineralocorticoid receptor antagonist (MRA) therapy has been shown in randomized trials to reduce mortality in patients with heart failure and reduced ejection fraction (HFrEF), MRA therapy is greatly underused in everyday practice (1, 2). Despite the evidence from clinical trials and a Class I, Level of Evidence: A recommendation in guidelines, registry data from different regions of the world consistently show lower use of MRA drugs than of angiotensin-converting enzyme (ACE) inhibitors/angiotensin receptor blockers (ARBs), or beta-blockers (3, 4). Hyperkalemia is a well-recognized concern, leading to underprescription of MRA drugs, but physicians also report an unwillingness to prescribe these medications in patients with low blood pressure (5). The findings of the recent PATHWAY-2 (Prevention And Treatment of Hypertension With Algorithm based therapY) trial seem to have led to the perception that MRAs are powerful antihypertensive agents and amplified the concern about hypotension in patients with HFrEF (6). In PATHWAY-2, spironolactone therapy started at 25 mg daily and force-titrated to 50 mg reduced home systolic blood pressure (SBP) by a mean of 8.70 mm Hg (95% confidence interval [CI]: −9.72 to −7.69; p < 0.0001) compared with placebo and was more effective than alternative fourth-line drugs (bisoprolol or doxazosin) in patients with resistant hypertension already treated with an ACE inhibitor/ARB, a calcium channel blocker, and a thiazide or thiazide-like diuretic.

To determine whether physicians should be concerned about MRA-induced hypotension in patients with HFrEF, this study analyzed the effect of MRA therapy on blood pressure and outcomes, according to baseline blood pressure in the 2 major randomized placebo-controlled trials using drugs in this class (spironolactone and eplerenone) in patients with HFrEF.

## Methods

### Details of trials included

The design, baseline findings, and primary results of the 2 trials have been reported previously in detail (1,2,7,8). Participants in each trial provided written informed consent. Briefly, the RALES (Randomized Aldactone Evaluation Study) was an event-driven, double-blind, placebo-controlled mortality trial. Patients with New York Heart Association (NYHA) functional classes III-IV heart failure with a ventricular ejection fraction (LVEF) of ≤35% were randomly assigned to receive placebo or spironolactone therapy. The starting dosage of the study drug was 25 mg of spironolactone once daily or matching placebo. After 8 weeks, the dosage could be increased to 50 mg daily if the patient still had symptoms of heart failure but did not have hyperkalemia.

The EMPHASIS-HF (Eplerenone in Mild Patients Hospitalization and Survival Study in Heart Failure) trial was an event-driven, double-blind, placebo-controlled trial with a composite morbidity-mortality outcome (cardiovascular death or heart failure hospitalization). Patients with NYHA functional class II heart failure with an LVEF of ≤35% were randomly assigned to placebo or eplerenone therapy. The starting dosage for the study drug was eplerenone 25 mg once daily or matching placebo. After 4 weeks, the dosage was increased to 50 mg once daily, unless there was hyperkalemia (or another intolerance).

Importantly, neither trial had a lower blood pressure exclusion criterion. The median follow-up in RALES was 24 months and 21 months in EMPHASIS-HF. In order to have an adequate number of patients (and events) in each baseline blood pressure category of interest (see below) and to cover the full spectrum of symptom severity (NYHA functional class II to IV), the RALES and EMPHASIS-HF databases were merged for analyses.

### Baseline blood pressure categories and serial blood pressure assessments

Patients were divided into 5 baseline SBP categories: ≤105, >105 to ≤115, >115 to ≤125, >125 to ≤135, and >135 mm Hg, to ensure equally sized groups across the spectrum of blood pressure values. In RALES, SBP was measured at 1, 2, 3, 6, and 9 months and at 1, 5, and 9 months in EMPHASIS-HF. For the purpose of this analysis, the 5- and 6-month measurements were considered to have occurred at 6 months.

### Study outcomes

The primary outcome used in this analysis was the composite of cardiovascular death or heart failure hospitalization. The components of this composite and all-cause death were also examined. Furthermore, we analyzed the occurrence of investigator-reported hypotension, a decrease in SBP ≥30 mm Hg at the 6-month measurement, decrease of SBP <85 mm Hg at the 1-month, 6-month and both measurements, elevation of serum potassium (>5.5 mmol/l and >6.0 mmol/l), elevation of serum creatinine (≥2.5 mg/dl and ≥3.0 mg/dl) and the rate of permanent study drug discontinuation for any reason other than death during the study follow-up.

### Statistical analysis

Summary statistics for baseline characteristics (including background treatment) are provided for each SBP category (Table 1). Continuous variables are shown as mean ± SD and categorical variables as frequencies and proportions. Baseline variables, the occurrence of permanent study drug discontinuation, and adverse effects were compared across SBP categories by using ANOVA for continuous variables and chi-square tests for categorical variables. The outcomes of interest across SBP categories were illustrated using Kaplan-Meier analysis. Cox proportional hazards models were used to estimate the hazard ratio for the effect of MRA treatment in each SBP category and to calculate the p value for the interaction between SBP category and effect of treatment. The Cox regression models included baseline age, sex, race, NYHA functional class, cause by ischemia, history of hypertension, diabetes, history of angina, history of myocardial infarction, heart rate, LVEF, serum potassium and serum creatinine concentrations, estimated glomerular filtration rate category (±60 ml/min/1.73 m^2^), baseline use of beta blockers, and digoxin. Restricted cubic spline analysis was used to examine the effect of treatment according to baseline SBP modeled as a continuous variable. The interaction between SBP as a continuous variable and treatment, on the occurrence of the composite outcome, its components, and all-cause death, was tested using an adjusted Cox regression model. The interaction between SBP category and treatment with reference to the occurrence of adverse events and withdrawal from study drug was tested using a logistic regression model with a term for interaction between SBP category and treatment. Changes in SBP were assessed by using a repeated measures mixed model with the baseline SBP as a covariate and treatment, time, and treatment by time interactions as fixed effects. Sensitivity analysis was performed, analyzing baseline characteristics, and rate of study outcome in the 2 trial populations separately (complete analyses are included in Online Tables S1 to S11, Online Figures S1 to S3). All statistical analyses were performed using the Stata/SE version 15.1 software (Stata Corp, College Station, Texas). All p values are 2-sided, and a p value of <0.05 was considered significant.

**Table 1:** Baseline Characteristics of Patients, Overall, and in Each SBP Category

	SBP (mm Hg)						p Value
	Overall (N = 4,396)	≤105 (n = 702)	>105 to ≤115 (n = 870)	>115 to ≤125 (n = 960)	>125 to ≤135 (n = 823)	>135 (n = 1,041)	
SBP, mm Hg	123.4 ± 18.2	97.6 ± 5.8	110.8 ± 2.2	120.8 ± 2.2	130.6 ± 2.1	148.0 ± 11.4	**<0.001**
DBP, mm Hg	74.6 ± 10.8	64.0 ± 8.0	69.6 ± 7.9	74.4 ± 7.9	77.9 ± 7.8	83.5 ± 10.3	**<0.001**
Age, yrs	67.3 ± 9.6	65.5 ± 10.6	66.2 ± 10.5	67.5 ± 9.3	68.2 ± 8.7	68.6 ± 8.8	**<0.001**
Females	1,056 (24.0)	163 (23.2)	190 (21.8)	226 (23.5)	180 (21.9)	297 (28.5)	**0.003**
Race							**<0.001**
White	3,705 (84.3)	553 (78.8)	716 (82.3)	831 (86.6)	718 (87.2)	887 (85.2)	
Black	187 (4.3)	46 (6.6)	43 (4.9)	29 (3.0)	20 (2.4)	49 (4.7)	
Asian	347 (7.9)	68 (9.7)	76 (8.7)	61 (6.4)	60 (7.3)	82 (7.9)	
Other	157 (3.6)	35 (5.0)	35 (4.0)	39 (4.1)	25 (3.0)	23 (2.2)	
NYHA functional class							**<0.001**
I	1 (<0.1)	0 (0.0)	0 (0.0)	1 (0.1)	0 (0.0)	0 (0.0)	
II	2,741 (62.4)	373 (53.1)	517 (59.4)	631 (65.7)	566 (68.8)	654 (62.8)	
III	1,171 (26.6)	205 (29.2)	249 (28.6)	233 (24.3)	187 (22.7)	297 (28.5)	
IV	483 (11.0)	124 (17.7)	104 (12.0)	95 (9.9)	70 (8.5)	90 (8.6)	
Ischemic cause	2,792 (63.6)	406 (57.9)	536 (61.6)	627 (65.3)	552 (67.2)	671 (64.6)	**0.001**
Hypertension	2,208 (50.2)	201 (28.6)	358 (41.1)	497 (51.8)	489 (59.4)	663 (63.7)	**<0.001**
Diabetes	1,227 (27.9)	158 (22.5)	218 (25.1)	243 (25.4)	266 (32.3)	342 (32.9)	**<0.001**
Previous angina	1,297 (29.5)	145 (20.7)	225 (25.9)	300 (31.3)	294 (35.7)	333 (32.0)	**<0.001**
Previous MI	1,852 (42.1)	275 (39.2)	364 (41.8)	433 (45.2)	365 (44.3)	415 (39.9)	**0.039**
Heart rate, beats/min	75.2 ± 13.9	76.4 ± 14.0	75.4 ± 14.1	74.4 ± 13.6	74.9 ± 13.2	75.2 ± 14.3	0.058
LVEF, %	25.8 ± 5.6	24.2 ± 6.5	25.4 ± 5.7	25.9 ± 5.3	26.4 ± 5.2	26.8 ± 5.0	**<0.001**
Potassium, mg/dl	4.28 ± 0.44	4.24 ± 0.44	4.26 ± 0.43	4.30 ± 0.45	4.30 ± 0.44	4.29 ± 0.42	**0.019**
Creatinine, mg/dl	1.18 ± 0.33	1.24 ± 0.34	1.18 ± 0.34	1.18 ± 0.33	1.16 ± 0.31	1.16 ± 0.33	**<0.001**
eGFR <60 ml/min/1.73 m^2^	1,699 (38.7)	316 (45.1)	332 (38.2)	382 (39.8)	282 (34.3)	387 (37.2)	**<0.001**
Diuretics	3,826 (87.3)	629 (89.6)	765 (88.2)	843 (88.0)	697 (85.2)	892 (86.1)	0.06
ACE inhibitor or ARB	4,144 (94.6)	655 (93.3)	816 (94.1)	914 (95.4)	781 (95.5)	978 (94.4)	0.27
Beta-blockers	2,545 (58.1)	350 (49.9)	492 (56.7)	576 (60.1)	530 (64.8)	597 (57.6)	**<0.001**
Digoxin	1,954 (44.6)	381 (54.3)	413 (47.6)	411 (42.9)	325 (39.7)	424 (40.9)	**<0.001**

Values are mean ± SD or n (%). The p values in **bold** indicate statistical significance.

ACE = angiotensin converting enzyme inhibitor; ARB = angiotensin receptor blocker; DBP = diastolic blood pressure; eGFR = estimated glomerular filtration rate; MI = myocardial infarction; NYHA = New York Heart Association; LVEF = left ventricular ejection fraction; SBP = systolic blood pressure.

## Results

Overall, 4,396 patients were included in the analysis, of whom 2,214 were randomized to placebo and 2,182 to an MRA. There were 702 patients with a baseline SBP of ≤105 mm Hg (a mean SBP of 97.6 ± 5.8 mm Hg); 870 patients with an SBP of >105 to ≤115 mm Hg (a mean SBP of 110.8 ± 2.2 mm Hg); 960 patients with an SBP of >115 to ≤125 mm Hg (a mean SBP of 120.8 ± 2.2 mm Hg); 823 patients with an SBP of >125 and ≤135 mm Hg (a mean SBP of 130.6 ± 2.1 mm Hg); and 1,041 patients with an SBP of >135 mm Hg (mean SBP of 148 ± 11.4 mm Hg).

### Baseline characteristics

The baseline characteristics according to SBP category are shown in Table 1. Patients with a lower SBP were younger, more often male, and had worse NYHA functional class status and lower median LVEF values. However, participants with a lower SBP were less likely to have a history of hypertension, coronary heart disease, or diabetes. They had worse renal function and a slightly lower potassium level than patients with a higher SBP. Patients in the lowest SBP category were least likely to be treated with a beta-blocker and more likely to be treated with a diuretic drug and digoxin. Use of an ACE inhibitor/ARB was similar across the SBP categories.

### Change in blood pressure

Overall, the mean change in SBP from baseline to 1 month was −0.5 ± 15.9 mm Hg in the placebo group and −2.4 ± 16.3 mm Hg in the MRA group, resulting in a between-treatment difference of 1.9 mm Hg (95% CI: 0.9 to 2.9; p < 0.001). The corresponding values at 6 months were +1.4 ± 18.1 mm Hg in the placebo group and −1.2 ± 17.9 mm Hg in the MRA group, giving a difference of 2.6 mm Hg (95% CI: 1.5 to 3.6; p < 0.001) and +0.9 ± 17.8 mm Hg in the placebo group and −0.8 ± 18.0 mm Hg in the MRA group at 9 months; giving a difference of 1.8 mm Hg (95% CI: 0.7 to 2.8; p = 0.002).

**Figure 1:**
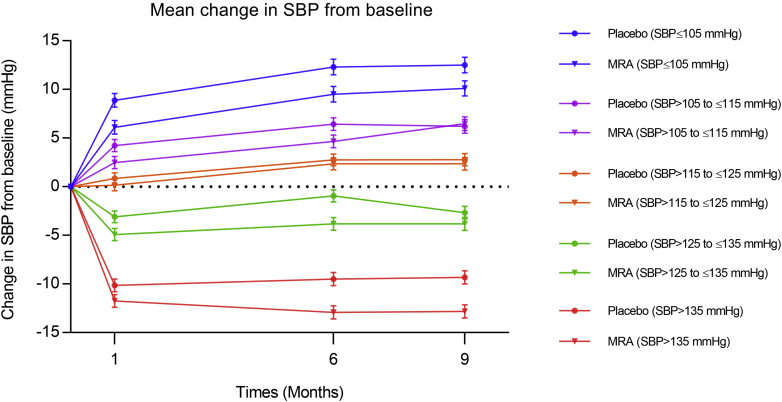
Mean Change in SBP Mean change in SBP from baseline to 1, 6, and 9 months in the placebo and in the MRA group in each baseline SBP category. MRA = mineralocorticoid receptor antagonist; SBP = systolic blood pressure.

Figure 1 shows the mean change in SBP from baseline during the first 9 months of treatment in each of the SBP categories of interest. These changes are also enumerated in Table 2. SBP increased in the patients with the lowest starting SBP level and decreased in those with a higher SBP at baseline. Among patients in the lower SBP categories, SBP increased less in MRA treated patients than in placebo-treated patients. In patients in the higher SBP categories, SBP decreased more with MRA therapy than in those undergoing placebo treatment.

**Table 2:** Change in Mean SBP From Baseline to 1 Month and to 6 Months and Between-Treatment Differences in SBP, Overall, and in Each SBP Category

Baseline SBP Category	Baseline to 1 Month			Baseline to 6 Months		
	Placebo	MRA	Difference	Placebo	MRA	Difference
≤105 mm Hg	8.9 ± 15.1	6.1 ± 13.5	2.8	12.3 ± 16.2	9.6 ± 15.1	2.8
>105 to ≤115 mm Hg	4.2 ± 13.3	2.5 ± 14.5	1.7	6.6 ± 16.4	4.8 ± 15.9	1.7
>115 to ≤125 mm Hg	0.9 ± 13.1	0.2 ± 14.5	0.7	2.9 ± 15.0	2.4 ± 15.8	0.4
>125 to ≤135 mm Hg	−3.1 ± 14.3	−4.9 ± 13.4	1.8	−0.9 ± 16.4	−3.7 ± 14.0	2.9
>135 mm Hg	−10.0 ± 16.6	−11.9 ± 17.7	1.8	−9.3 ± 18.6	−13.0 ± 18.0	3.6
Overall	−0.5 ± 15.9	−2.4 ± 16.3	1.9	1.4 ± 18.1	−1.2 ± 17.9	2.6

Values are mean ± SD changes in SBP.

MRA = mineralocorticoid receptor antagonist; SBP = systolic blood pressure.

### Clinical outcomes

**Figure 2:**
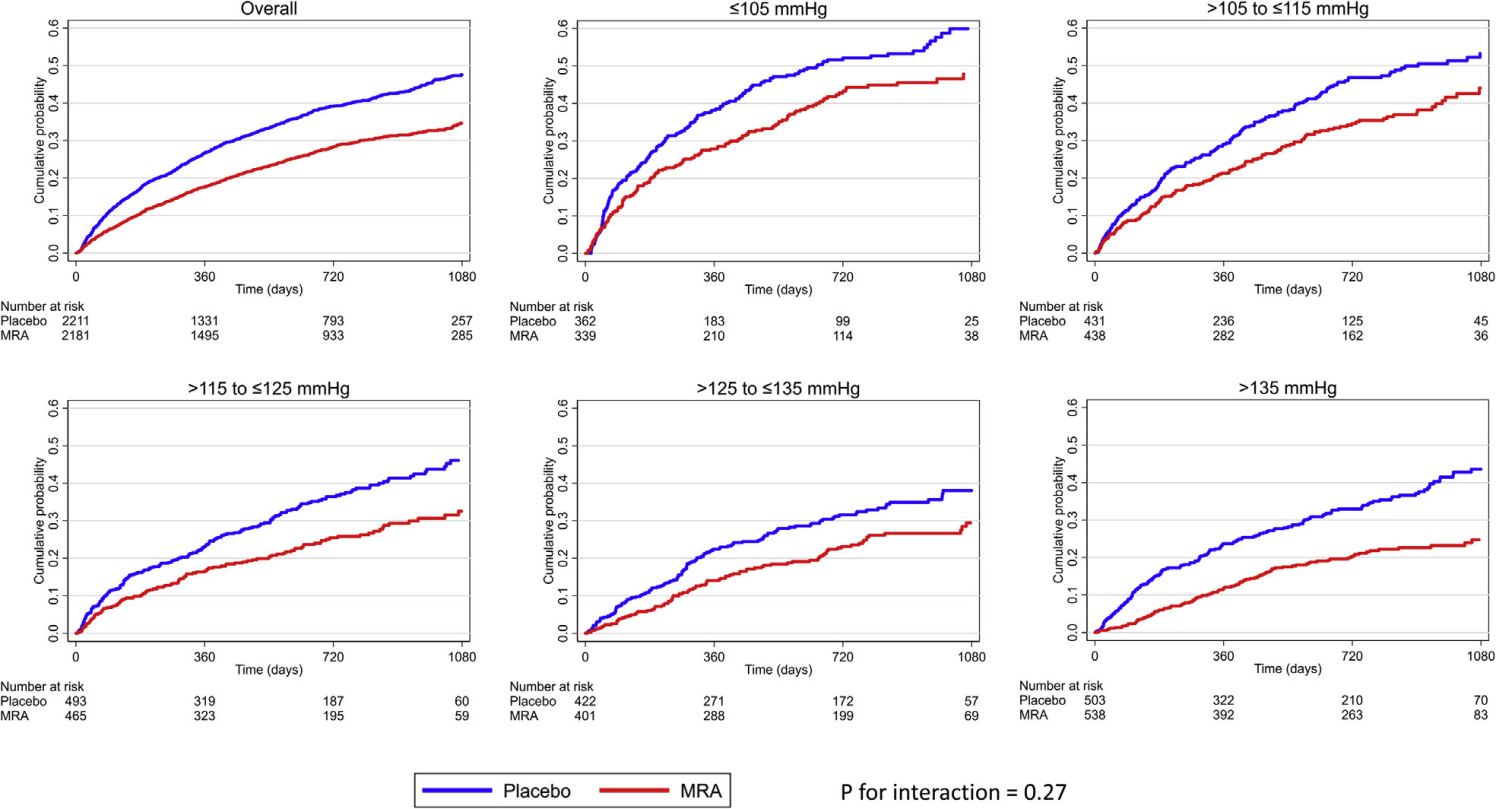
Cumulative Incidence of Cardiovascular Death or Hospitalization for Heart Failure According to Baseline SBP Categories Kaplan-Meier curves for cardiovascular death or hospitalization for heart failure, overall, and in each SBP category. The p value is for interaction between baseline SBP categories and treatment effect. The risk table below the graphs shows the number at risk of the event of interest. MRA denotes mineralocorticoid receptor antagonist. Abbreviations as in Figure 1.

**Figure 3:**
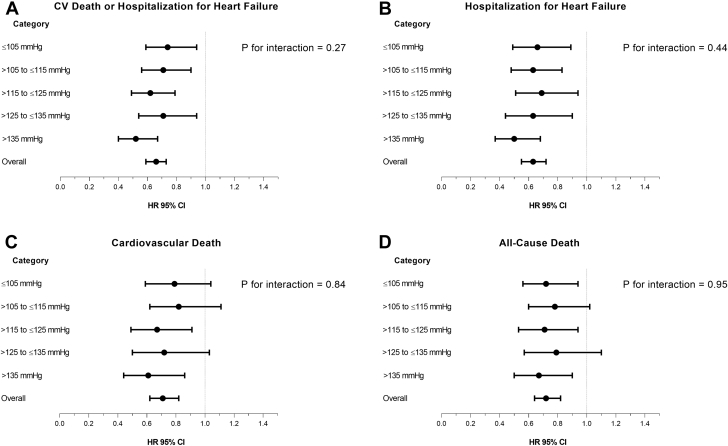
Adjusted HR for Clinical Outcomes According to SBP Categories **(A)** Adjusted HR for cardiovascular death or hospitalization for heart failure, **(B)** hospitalization for heart failure, **(C)** cardiovascular death, and **(D)** all-cause death overall, and in each SBP category. The p values are for interaction between baseline SBP categories and treatment effect. CI = confidence interval, HR = hazard ratio; other abbreviations as in Figure 1.

Examination of the placebo group showed that the rates of all outcomes of interest were higher in patients with a lower baseline SBP (Table 3, Figure 2). Overall, compared with placebo, MRA therapy reduced the risk of the primary composite outcome (cardiovascular death or heart failure hospitalization), its components, and all-cause death (Figures 2 and 3). The benefit of MRA therapy was consistent across SBP categories, and there was no evidence that baseline SBP modified the effect of MRA therapy (the SBP-treatment interaction test result was nonsignificant for each outcome of interest) (Table 3, Figure 3). Similar findings were observed examining pretreatment SBP as a continuous variable (Online Figure S1).

**Table 3:** Effect of MRA Treatment on Clinical Outcomes Overall, and in Each SBP Category

Outcome	Number of Events (%)			Adjusted HR (95% CI), p Value	p Value for Interaction∗
	Overall	Placebo	MRA		
CV death or HF hospitalization					0.27
≤105 mm Hg	312 (44.4)	176 (48.5)	136 (40.1)	0.74 (0.59–0.94), 0.012	
>105 to ≤115 mm Hg	319 (36.7)	177 (41.1)	142 (32.2)	0.71 (0.56–0.90), 0.004	
>115 to ≤125 mm Hg	293 (30.5)	177 (35.8)	116 (24.9)	0.62 (0.49–0.79), <0.001	
>125 to ≤135 mm Hg	222 (27.0)	129 (30.6)	93 (23.2)	0.71 (0.54–0.94), 0.015	
>135 mm Hg	275 (26.4)	169 (33.6)	106 (19.7)	0.52 (0.40–0.67), <0.001	
Overall	1,421 (32.35)	828 (37.4)	593 (17.2)	0.66 (0.59–0.73), <0.001	
Heart failure hospitalization					0.44
≤105 mm Hg	192 (27.4)	113 (31.1)	79 (23.3)	0.66 (0.49–0.89), 0.006	
>105 to ≤115 mm Hg	227 (26.1)	131 (30.4)	96 (21.9)	0.63 (0.48–0.83), 0.001	
>115 to ≤125 mm Hg	189 (19.7)	110 (22.2)	79 (17.0)	0.69 (0.51–0.94), 0.017	
>125 to ≤135 mm Hg	139 (16.9)	84 (19.9)	55 (13.7)	0.63 (0.44–0.90), 0.010	
>135 mm Hg	185 (17.8)	115 (22.9)	70 (13.0)	0.50 (0.37–0.68), <0.001	
Overall	932 (21.2)	553 (25.0)	379 (17.4)	0.63 (0.55–0.72), <0.001	
Cardiovascular death					0.84
≤105 mm Hg	216 (30.8)	119 (32.8)	97 (28.6)	0.79 (0.59–1.04), 0.092	
>105 to ≤115 mm Hg	200 (23.0)	110 (25.5)	90 (20.5)	0.82 (0.62–1.11), 0.197	
>115 to ≤125 mm Hg	178 (18.5)	107 (21.6)	71 (15.3)	0.67 (0.49–0.91), 0.010	
>125 to ≤135 mm Hg	131 (15.9)	76 (18.0)	55 (13.7)	0.72 (0.50–1.03), 0.076	
>135 mm Hg	145 (13.9)	87 (17.3)	58 (10.8)	0.61 (0.44–0.86), 0.005	
Overall	870 (19.8)	499 (22.5)	371 (17.0)	0.71 (0.62–0.82), <0.001	
All-cause death					0.95
≤105 mm Hg	253 (36.0)	144 (39.7)	109 (32.2)	0.72 (0.56–0.94), 0.015	
>105 to ≤115 mm Hg	243 (27.9)	135 (31.3)	108 (24.6)	0.78 (0.60–1.02), 0.073	
>115 to ≤125 mm Hg	214 (22.3)	125 (25.3)	89 (19.1)	0.71 (0.53–0.94), 0.016	
>125 to ≤135 mm Hg	154 (18.7)	86 (20.4)	68 (17.0)	0.79 (0.57–1.10), 0.163	
>135 mm Hg	188 (18.1)	109 (21.7)	79 (14.7)	0.67 (0.50–0.90), 0.009	
Overall	1,052 (23.9)	599 (27.1)	453 (20.8)	0.72 (0.64–0.82), <0.001	

Values are n (%), unless otherwise noted.

CI = confidence interval; CV = cardiovascular; HF = heart failure; HR = hazard ratio; MRA = mineralocorticoid receptor antagonist; SBP = systolic blood pressure.

∗ The p values indicate interactions between SBP categories and treatment effects.

### Adverse effects and study drug discontinuation

Overall, 186 patients (4.2%) experienced hypotension, 86 (3.9%) in the placebo group and 100 (4.6%) in the MRA group (p = 0.25) (Table 4). The rate of hypotension in patients treated with placebo was highest in the lowest SBP group: 32 (8.8%) in those with a SBP ≤105 mm Hg, 17 (3.9%) for SBP of 105 to 115 mm Hg, 16 (3.2%) for SBP of 115 to 125 mm Hg, 13 (3.1%) for SBP of 125 to 135 mm Hg, and 8 (1.6%) in those with SBP > 135 mm Hg. The corresponding numbers/proportions in MRA treated patients were 35 (10.3%), 18 (4.1%), 22 (4.7%), 9 (2.2%), and 16 (3.0%), respectively. There was no interaction between SBP category and the effect of treatment, with respect to the occurrence of hypotension (Online Table S1).

**Table 4:** Adverse Effects of Interest and Permanent Study Drug Discontinuation

Event	Number of Adverse Effects (%)			
	Overall (N = 4,396)	Placebo (n = 2,214)	MRA (n = 2,182)	p Value
Hypotension	186 (4.2)	86 (3.9)	100 (4.6)	0.25
SBP drop ≥30 mm Hg by 6 months	339 (7.7)	150 (6.8)	189 (8.7)	**0.019**
SBP <85 mm Hg at 1 month∗	37 (0.8)	16 (0.7)	21 (1.0)	0.39
SBP <85 mm Hg at 6 months∗	29 (0.7)	12 (0.5)	17 (0.8)	0.33
SBP <85 mm Hg at 1 and 6 months∗	5 (0.1)	2 (0.1)	3 (0.1)	0.64
Creatinine ≥2.5 mg/dl	163 (3.7)	65 (2.9)	98 (4.5)	**0.006**
Creatinine ≥3.0 mg/dl	67 (1.5)	28 (1.3)	39 (1.8)	0.16
Potassium >5.5 mmol/l	419 (9.5)	132 (6.0)	287 (13.2)	**<0.001**
Potassium >6.0 mmol/l	98 (2.2)	32 (1.4)	66 (3.0)	**<0.001**
Discontinuation of study drug	876 (19.9)	441 (19.9)	435 (19.9)	0.99

Values are n (%). The p values in **bold** indicate statistical significance.

Abbreviations as in Tables 1 and 2.

∗ Patients with baseline SBP lower than 85 mm Hg were excluded from this analysis.

Overall, 37 patients (0.8%) experienced a decrease in SBP <85 mm Hg at the 1-month measurement, 16 (0.7%) in the placebo group and 21 (1.0%) in the MRA group (p = 0.39). At the 6-month measurement, 29 patients (0.7%) showed a decrease in SBP <85 mm Hg, 12 (0.5%) in the placebo group and 17 (0.8%) in the MRA group (p = 0.33). Only 5 patients (0.1%) experienced an SBP <85 mm Hg at both the 1-month and the 6-month assessments, 2 (0.1%) in the placebo group and 3 (0.1%) in the MRA group (p = 0.64).

Elevation of creatinine and potassium concentrations was more common in the MRA than in the placebo group (Table 4), although the rate of these adverse effects did not differ greatly across SBP categories (Online Table S1).

Overall, 876 patients (19.9%) permanently discontinued study treatment for reasons other than death, 441 (19.9%) in the placebo group and 435 (19.9%) in the MRA group (p = 0.99) (Table 4). The overall number and rate of study drug discontinuation in the placebo group was 85 (23.4%) in those with an SBP of ≤105 mm Hg, 96 (22.3%) with an SBP of >105 to ≤115 mm Hg, 86 (17.4%) with an SBP of >115 to ≤125 mm Hg, 82 (19.4%) with an SBP of >125 to ≤135 mm Hg, and 92 (18.3%) in those with an SBP of >135 mm Hg. The corresponding numbers and proportions in MRA-treated patients were 72 (21.2%), 98 (22.3%), 88 (18.9%), 64 (16.0%), and 113 (21.0%), respectively (Online Table S1). The rates of discontinuation between the placebo and MRA groups did not differ across SBP categories.

## Discussion

Neither RALES nor EMPHASIS-HF had a lower SBP exclusion criterion, unlike most prior trials in patients with HFrEF (1,2). Accordingly, 702 of the 4,396 patients (16%) in the present study had an SBP of ≤105 mm Hg. In keeping with most previous studies, the present study found that patients with a low SBP had worse outcomes, despite being younger and having less comorbidity than patients with a higher SBP; however, patients with lower SBP did have more advanced NYHA functional class, more renal impairment, and lower LVEF values (9, 10, 11).

The 2 major new findings of the present analyses were that MRA treatment had little effect on blood pressure in patients with HFrEF, in contrast to the case in hypertension, and that the beneficial effect of MRA therapy was not modified by baseline SBP (Central Illustration).

**Central Illustration:**
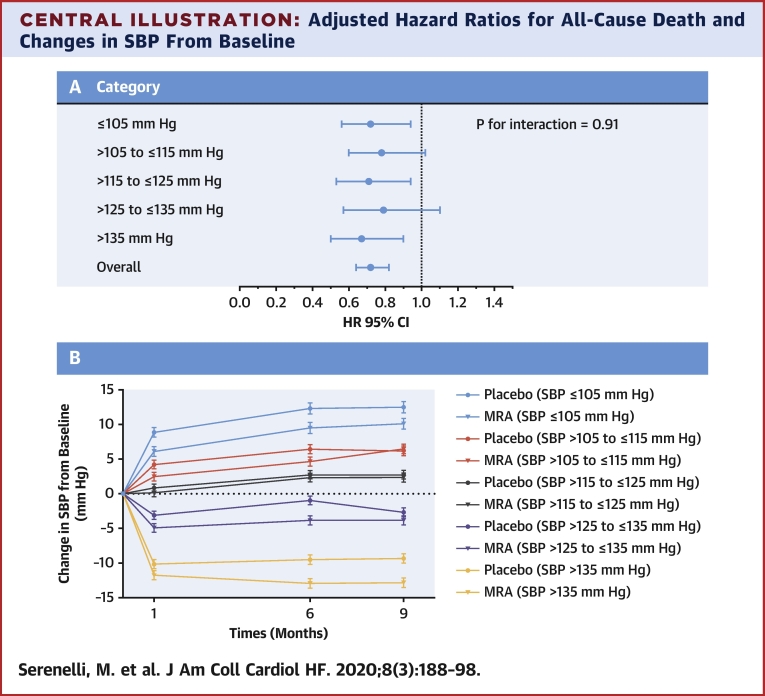
Adjusted Hazard Ratios for All-Cause Death and Changes in SBP From Baseline **(A)** Adjusted hazard ratios for all-cause death and **(B)** the mean change in SBP from baseline to 1, 6, and 9 months in each baseline SBP category. The treatment effect and the small blood pressure-lowering effect observed are consistent across the baseline SBP categories. The p value is for interaction between baseline SBP category and treatment effect. MRA = mineralocorticoid receptor antagonist; SBP = systolic blood pressure.

Overall, the differences in SBP between the placebo and the MRA therapy were small, namely 1.9 ± 0.5 mm Hg at 1 month and 2.6 ± 0.6 mm Hg at 6 months (and did not differ between spironolactone and eplerenone). It is instructive to compare this to the reduction in SBP in PATHWAY-2 patients with resistant hypertension (6). In PATHWAY-2, spironolactone was started at 25 mg daily for 6 weeks and then force-titrated to 50 mg daily for a further 6 weeks (total treatment duration of 12 weeks). In patients who received both doses of each tested drug, the overall mean placebo-corrected reduction in home SBP with spironolactone was 9.15 mm Hg (at 6 weeks it was 6.86 mm Hg and 11.4 mm Hg at 12 weeks). The corresponding mean overall placebo-corrected reduction in clinic SBP was 9.92 mm Hg (separate 6- and 12-week data were not reported). It is important to note that, because of this sequential design, it was not possible to tell how much of the incremental SBP reduction, if any, was due to the increase in dose of spironolactone. Also, the average dose of spironolactone attained was not reported in PATHWAY-2. It is likely, however, that the proportion of patients achieving the target dose of spironolactone in PATHWAY-2 was greater than the proportion achieving the target dose of MRA in the 2 HFrEF trials, which had a similar forced-titration design. In EMPHASIS-HF, only 60.2% of patients assigned to eplerenone therapy were receiving 50 mg daily at the end of the titration phase, and the mean daily dose of eplerenone was 39.1 mg (at 5 months). Information for mean doses of spironolactone achieved during the first year of follow-up in RALES was not available.

There are additional explanations, other than dose, for the differences in SBP reduction between these trials. One explanation is that PATHWAY-2 enrolled patients with a higher mean SBP (148 mm Hg) than in the present patients with HFrEF (123 mm Hg), and generally, patients with higher starting SBP show a greater reduction with treatment than those with a lower baseline SBP (see below). However, the patients in PATHWAY-2 had resistant hypertension, and the reductions in SBP with the other active therapies tested (bisoprolol and doxazosin) were approximately one-half that obtained with spironolactone.

Alternatively, the PATHWAY-2 investigators proposed that patients in their trial were especially responsive to spironolactone because the major pathophysiological cause of resistant hypertension is sodium retention; however, this is also characteristic of patients with HFrEF, yet in those patients, the reduction in SBP with MRA treatment was small. It has also been suggested that spironolactone is particularly effective in patients with resistant hypertension because many have increased aldosterone secretion; however, this is also true of patients with heart failure.

Although there may be no clear explanation for the different SBP responses to MRA treatment in resistant hypertension and HFrEF, differential SBP responses to other therapies in hypertension and heart failure have been reported. For example, in the second Cardiac Insufficiency Bisoprolol Study (12), where patients were titrated to a target dosage of bisoprolol, 10 mg once daily, the placebo-corrected mean reduction in SBP from baseline to 3 months was 1.37 (95% CI: −4.81 to 2.08) mm Hg (M Serenelli and J McMurray, January 2020). This compares with a reduction in home SBP at 3 months in the PATHWAY-2 study of 4.57 (95% CI: −5.60 to −3.54) mm Hg with the same beta-blocker, titrated to the same daily dose. Consistent with this was the reduction in SBP of 1.98 mm Hg at 6 weeks and 4.55 mm Hg at 6 months with candesartan titrated to a dosage of 32 mg daily in the CHARM-Alternative (Candesartan in Heart failure: Assessment of Reduction in Mortality and morbidity-Alternative) trial (13) (M Serenelli and J McMurray, January 2020), compared to a reduction of 10.5 mm Hg after 8 weeks with the dosing approach in patients with hypertension (14). In other words, there is good evidence that the SBP decrease in response to 3 different classes of drugs is different in HFrEF than in hypertension. Although the explanation why is uncertain, there is an important lesson here for health care providers who may be concerned about giving drugs considered to be “antihypertensives” to patients with HFrEF.

As in other HFrEF trials examining the effect of different heart failure medications on SBP, patients in RALES and EMPHASIS-HF with the lowest baseline SBP experienced an increase in SBP after randomization in both treatment groups, although the increase was smaller in the MRA group (10, 15, 16, 17, 18). In contrast, patients with higher SBP experienced a decrease in SBP after randomization, although more so with MRA treatment than with placebo. This likely reflects the phenomenon of “regression to the mean.”

It is also of interest to compare the present findings in patients with HFrEF with those with spironolactone in HF with preserved EF (HFpEF), where patients often have a hypertensive background and usually have a higher SBP than individuals with HFrEF. In patients from North and South America enrolled in the TOPCAT (Treatment of Preserved Cardiac Function Heart Failure with an Aldosterone Antagonist) trial, baseline SBP was 129 mm Hg. At 4 months, the change from baseline was +0.94 ± SE 0.6) mm Hg in the placebo group and −2.75 ± SE 0.6, giving a difference of −3.69 ± SE 0.86 mm Hg (p < 0.001) (19).

In the present study, patients in the lowest SBP group had a similar relative risk reduction in all outcomes of interest with MRA treatment, as patients with a higher SBP. This is relevant because many physicians are concerned about prescribing drugs with a hypotensive effect to heart failure patients with low blood pressure, potentially depriving them of the mortality benefit of these treatments (20). Indeed, applying the overall relative risk reduction of 27% in all-cause mortality with MRA treatment, patients in the lowest SBP category would have the greatest absolute mortality benefit because they had the highest absolute mortality rate. Specifically, treatment would lead to approximately 11 fewer deaths per 100 patients treated with an MRA drug in the SBP group of ≤105 mm Hg, compared with 6 fewer deaths per 100 patients in the SBP group of >135 mm Hg.

In keeping with the SBP change data reported above, the rate of hypotension with MRA therapy, assessed either by SBP measurement or adverse effect reporting, was low. As expected, the highest rate of hypotension was reported in the lowest SBP categories, but this was the case with both placebo and MRA treatment. The placebo-corrected difference in rates of hypotension was similar across SBP categories, and the difference between randomized treatments was not significant in any SBP category. Moreover, the rates of severe renal dysfunction (creatinine concentration of ≥3.0 mg/dl) and of discontinuation of study drug were the same in the 2 treatment groups.

### Study limitations

First, it is retrospective, and the analyses reported were not planned prospectively. The measurement of blood pressure was not standardized, which will have increased imprecision around the values reported. Aldosterone levels, which might have helped to understand the effects of MRA treatment on SBP were not measured. The proportions of patients treated with a beta-blocker at baseline were significantly different in the 2 trials. Rates of use of beta-blocker therapy in real world registries are approximately 80%, but no evidence was found that the blood pressure-lowering effects of MRA were different in the 2 trials, where beta-blocker use was different at baseline. The present results would therefore be applicable to the wider population where rates of beta-blocker use lie between these 2 extremes.

Finally, the effects of eplerenone, according to baseline SBP at or above versus below the median value (123 mm Hg), have previously been described in EMPHASIS-HF, although only the primary composite outcome was reported (21).

## Conclusions

MRA treatment was found to have little effect on SBP in patients with HFrEF, in contrast to the case in hypertension. The beneficial effect of MRA therapy on clinical outcomes in patients with HFrEF was not modified by baseline SBP. Because patients with HFrEF with the lowest SBP had the highest event rates, they had the greatest absolute benefit from MRA therapy. Hypotension was infrequently caused by MRA treatment, even in patients with a low baseline SBP. MRA therapy was discontinued at a rate similar to that of placebo overall and in patients with the lowest baseline SBP. Low SBP is not a reason to withhold MRA therapy in patients with HFrEF.

Perspectives **COMPETENCY IN MEDICAL KNOWLEDGE:** Despite strong guideline recommendations, registries show that many patients with HFrEF are not treated with an MRA. One reason for withholding this treatment is low SBP. However, the present analysis shows that MRAs have a small effect on SBP in patients with HFrEF, are well tolerated even in patients with HFrEF with a low SBP, and have a large absolute benefit in such individuals, as patients with a low SBP are at high risk.

**TRANSLATIONAL OUTLOOK:** Contrary to that found in hypertension, MRAs do not have a substantial SBP-lowering effect in HFrEF. This appears to be generally true of all therapies that are used as both antihypertensives and to treat HFrEF. Although the reasons for this difference in the effect of treatment on SBP in the 2 conditions are uncertain, it is important that health care providers are aware of it so that life-saving treatments are not withheld in HFrEF because of inappropriate concerns about hypotension.
