# Supplementary material for: Mineralocorticoid Receptor Antagonists, Blood Pressure, and Outcomes in Heart Failure With Reduced Ejection Fraction
Source: JACC Heart Fail. 2020 Mar;8(3):188–98. doi: 10.1016/j.jchf.2019.09.011 (PMC7086149; doi:10.1016/j.jchf.2019.09.011)
Supplement: Online Tables 1–11 and Online Figures 1–3 [file mmc1.docx]

**Index of tables and figures.**

**Table S1.** Adverse effects of interest and permanent study drug discontinuation according to SBP categories.

**Table S2.** Baseline characteristics of patients, overall and in each SBP category, in the EMPHASIS-HF trial population.

**Table S3.** Baseline characteristics of patients, overall and in each SBP category, in the RALES trial population.

**Table S4.** Mean change in SBP from baseline to 1 month and to 6 months, and between-treatment difference in SBP, overall and in each SBP category, in the EMPHASIS-HF trial population.

**Table S5.** Mean change in SBP from baseline to 1 month and to 6 months, and between-treatment difference in SBP, overall and in each SBP category in the RALES trial population.

**Table S6.** Effect of MRA treatment on clinical outcomes, overall and in each SBP category, in the EMPHASIS-HF trial population.

**Table S7**. Effect of MRA treatment on clinical outcomes, overall and in each SBP category, in the RALES trial population.

**Table S8.** Rate of adverse effects and permanent study drug discontinuation according to treatment in the EMPHASIS-HF trial population.

**Table S9.** Rate of adverse effects and permanent study drug discontinuation according to treatment in the RALES trial population.

**Table S10**. Rate of adverse effects and permanent study drug discontinuation according to treatment and SBP categories in the EMPHASIS-HF trial population.

**Table S11**. Rate of adverse effects and permanent study drug discontinuation according to treatment and SBP categories in the RALES trial population.

**Figure S1.** Hazard ratio for (A) Cardiovascular Death or First Hospitalization for Heart Failure, (B) Hospitalization for Heart Failure, (C) Cardiovascular Death and (D) All-Cause Death in patient treated with MRA, according to baseline SBP.

**Figure S2.** Hazard ratio for (A) Cardiovascular Death or First Hospitalization for Heart Failure, (B) Hospitalization for Heart Failure, (C) Cardiovascular Death and (D) All-Cause Death in patient treated with MRA, according to baseline SBP in the EMPHASIS-HF trial population.

**Figure S3.** Hazard ratio for (A) Cardiovascular Death or First Hospitalization for Heart Failure, (B) Hospitalization for Heart Failure, (C) Cardiovascular Death and (D) All-Cause Death in patient treated with MRA, according to baseline SBP in the RALES trial population.

**Table S1.** **Adverse effects of interest and permanent study drug discontinuation according to SBP categories.**

|  | Adverse Effects, n (%) | | | | | | | | | |  |
| --- | --- | --- | --- | --- | --- | --- | --- | --- | --- | --- | --- |
| Variable | **≤105 mmHg** | | **>105 to ≤115 mmHg** | | **>115 to ≤125 mmHg** | | **>125 to ≤135 mmHg** | | **≥135 mmHg** | | **P value** |
|  | **Placebo**  **(N = 363)** | **MRA**  **(N = 339)** | **Placebo**  **(N = 431)** | **MRA**  **(N = 439)** | **Placebo**  **(N = 495)** | **MRA**  **(N = 465)** | **Placebo**  **(N = 422)** | **MRA**  **(N = 401)** | **Placebo**  **(N = 503)** | **MRA**  **(N = 538)** |  |
| Hypotension | 32 (8.8) | 35 (10.3) | 17 (3.9) | 18 (4.1) | 16 (3.2) | 22 (4.7) | 13 (3.1) | 9 (2.2) | 8 (1.6) | 16 (3.0) | 0.54 |
| SBP drop ≥30 mmHg to 6 months | 0 (0.0) | 1 (0.3) | 3 (0.7) | 5 (1.1) | 5 (1.0) | 18 (3.9) | 27 (6.4) | 28 (7.0) | 115 (22.9) | 137 (25.5) | -^†^ |
| SBP <85 mmHg* at 1 month | 14 (4.0) | 13 (3.9) | 1 (0.2) | 5 (1.1) | 0 (0.0) | 2 (0.4) | 1 (0.2) | 1 (0.3) | 0 (0.0) | 0 (0.0) | -^†^ |
| SBP <85 mmHg* at 6 months | 9 (2.6) | 14 (4.2) | 2 (0.5) | 2 (0.5) | 0 (0.0) | 0 (0.0) | 0 (0.0) | 0 (0.0) | 1 (0.2) | 1 (0.2) | 0.83 |
| SBP <85 mmHg* at 1 month and 6 months | 2 (0.6) | 2 (0.6) | 0 (0.0) | 1 (0.2) | 0 (0.0) | 0 (0.0) | 0 (0.0) | 0 (0.0) | 0 (0.0) | 0 (0.0) | -^†^ |
| Creatinine ≥ 2.5 mg/dL | 13 (3.6) | 26 (7.7) | 15 (3.5) | 19 (4.3) | 15 (3.0) | 17 (3.7) | 7 (1.7) | 11 (2.7) | 15 (3.0) | 25 (4.6) | 0.74 |
| Creatinine ≥ 3.0 mg/dL | 6 (1.7) | 15 (4.4) | 6 (1.4) | 4 (0.9) | 6 (1.2) | 4 (0.9) | 2 (0.5) | 5 (1.2) | 8 (1.6) | 11 (2.0) | 0.26 |
| Potassium > 5.5 mmol/L | 15 (4.1) | 36 (10.6) | 27 (6.3) | 48 (10.9) | 28 (5.7) | 67 (14.4) | 24 (5.7) | 57 (14.2) | 38 (7.6) | 79 (14.7) | 0.66 |
| Potassium > 6.0 mmol/L | 4 (1.1) | 10 (2.9) | 8 (1.9) | 11 (2.5) | 7 (1.4) | 11 (2.4) | 3 (0.7) | 10 (2.5) | 10 (2.0) | 24 (4.5) | 0.74 |
| Discontinuation of study drug | 85 (23.4) | 72 (21.2) | 96 (22.3) | 98 (22.3) | 86 (17.4) | 88 (18.9) | 82 (19.4) | 64 (16.0) | 92 (18.3) | 113 (21.0) | 0.44 |

MRA denotes mineralocorticoid receptor antagonist, SBP systolic blood pressure. P-values are for interaction between systolic blood pressure categories and treatment effect.

*Patients with baseline SBP lower than 85 mmHg, were excluded from this analysis

†P value for interaction not provided because of few events

**Table S2. Baseline characteristics of patients, overall and in each SBP category, in the EMPHASIS-HF trial population.**

|  |  | SBP category, mmHg | | | | | |  |
| --- | --- | --- | --- | --- | --- | --- | --- | --- |
| Variable | **Statistic** | **Overall**  **(N=2736)** | **≤105**  **(N=372)** | **>105 to ≤115**  **(N=517)** | **>115 to ≤125**  **(N=628)** | **>125 to ≤135**  **(N=566)** | **>135**  **(N=653)** | **P value** |
| SBP (mmHg) | Mean (SD) | 124.1 (16.9) | 98.2 (5.5) | 110.9 (2.4) | 121.0 (2.4) | 130.7 (2.3) | 146.6 (9.6) | **<0.001** |
| DBP (mmHg) | Mean (SD) | 74.6 (10.2) | 64.0 (7.7) | 68.9 (7.6) | 74.2 (7.5) | 77.9 (7.6) | 82.8 (9.4) | **<0.001** |
| Age (years) | Mean (SD) | 68.6 (7.6) | 67.9 (7.3) | 68.4 (7.9) | 68.5 (7.8) | 68.8 (7.6) | 69.3 (7.4) | **0.046** |
| Female | N (%) | 610 (22.3) | 74 (19.9) | 116 (22.4) | 133 (21.2) | 121 (21.4) | 166 (25.4) | 0.23 |
| Race |  |  |  |  |  |  |  | **<0.001** |
| White | N (%) | 2268 (82.9) | 285 (76.6) | 420 (81.2) | 532 (84.7) | 487 (86.0) | 544 (83.3) |  |
| Black | N (%) | 67 (2.4) | 16 (4.3) | 11 (2.1) | 16 (2.5) | 4 (0.7) | 20 (3.1) |  |
| Asian | N (%) | 315 (11.5) | 59 (15.9) | 70 (13.5) | 55 (8.8) | 56 (9.9) | 75 (11.5) |  |
| Other | N (%) | 86 (3.1) | 12 (3.2) | 16 (3.1) | 25 (4.0) | 19 (3.4) | 14 (2.1) |  |
| NYHA functional class |  |  |  |  |  |  |  |  |
| I | N (%) | 0 (0.0) | 0 (0.0) | 0 (0.0) | 0 (0.0) | 0 (0.0) | 0 (0.0) |  |
| II | N (%) | 2736 (100.0) | 372 (100.0) | 517 (100.0) | 628 (100.0) | 566 (100.0) | 653 (100.0) |  |
| III | N (%) | 0 (0.0) | 0 (0.0) | 0 (0.0) | 0 (0.0) | 0 (0.0) | 0 (0.0) |  |
| IV | N (%) | 0 (0.0) | 0 (0.0) | 0 (0.0) | 0 (0.0) | 0 (0.0) | 0 (0.0) |  |
| Ischemic etiology | N (%) | 1886 (69) | 238 (64.2) | 337 (65.2) | 442 (70.4) | 411 (72.9) | 458 (70.2) | **0.013** |
| Hypertension | N (%) | 1819 (66.5) | 155 (41.7) | 302 (58.4) | 417 (66.4) | 410 (72.4) | 535 (81.9) | **<0.001** |
| Diabetes | N (%) | 859 (31.4) | 107 (28.8) | 149 (28.8) | 184 (29.4) | 190 (33.6) | 229 (35.1) | **0.054** |
| Previous angina | N (%) | 1188 (43.5) | 127 (34.1) | 202 (39.1) | 284 (45.4) | 273 (48.2) | 302 (46.2) | **<0.001** |
| Previous MI | N (%) | 1380 (50.5) | 183 (49.2) | 261 (50.5) | 336 (53.7) | 288 (50.9) | 312 (47.8) | **0.31** |
| Heart Rate (bpm) | Mean (SD) | 71.7 (12.5) | 71.9 (11.9) | 71.5 (12.2) | 71.5 (12.1) | 72.0 (12.4) | 71.9 (13.3) | 0.91 |
| LVEF (%) | Mean (SD) | 26.1 (4.7) | 24.8 (5.6) | 25.9 (4.7) | 26.1 (4.6) | 26.5 (4.3) | 26.7 (4.3) | **<0.001** |
| Potassium (mg/dL) | Mean (SD) | 4.32 (0.43) | 4.27 (0.43) | 4.31 (0.40) | 4.34 (0.44) | 4.33 (0.44) | 4.32 (0.42) | 0.085 |
| Creatinine (mg/dL) | Mean (SD) | 1.15 (0.31) | 1.21 (0.32) | 1.16 (0.31) | 1.15 (0.30) | 1.13 (0.30) | 1.15 (0.30) | **<0.001** |
| eGFR<60 mL/min/1.73m^2^ | N (%) | 910 (33.2) | 149 (40.1) | 189 (36.6) | 213 (33.9) | 161 (28.4) | 198 (30.3) | **0.001** |
| Diuretics | N (%) | 2326 (85.5) | 330 (88.7) | 453 (88.1) | 540 (86.3) | 460 (82.0) | 543 (83.8) | **0.010** |
| ACEi or ARB | N (%) | 2558 (94.0) | 342 (91.9) | 473 (92.0) | 590 (94.2) | 539 (96.1) | 614 (94.8) | **0.022** |
| Betablockers | N (%) | 2374 (87.2) | 329 (88.4) | 453 (88.1) | 541 (86.4) | 499 (88.9) | 552 (85.2) | 0.27 |
| Digoxin | N (%) | 740 (27.2) | 110 (29.6) | 154 (30.0) | 176 (28.1) | 142 (25.3) | 158 (24.4) | 0.14 |

ACEi denotes angiotensin converting enzyme inhibitor, ARB angiotensin receptor blocker, DBP diastolic blood pressure, eGFR estimated glomerular filtration rate, MI myocardial infarction, NYHA New York heart association class, LVEF left ventricular ejection fraction, SBP systolic blood pressure.

**Table S3. Baseline characteristics of patients, overall and in each SBP category,** **in the RALES trial population.**

|  |  | SBP category, mmHg | | | | | |  |
| --- | --- | --- | --- | --- | --- | --- | --- | --- |
| Variable | **Statistic** | **Overall**  **(N=1660)** | **≤105**  **(N=330)** | **>105 to ≤115**  **(N=353)** | **>115 to ≤125**  **(N=332)** | **>125 to ≤135**  **(N=257)** | **>135**  **(N=388)** | **P**  **value** |
| SBP (mmHg) | Mean (SD) | 122.2 (20.1) | 97.0 (6.0) | 110.6 (1.8) | 120.5 (1.8) | 130.3 (1.6) | 150.4 (13.7) | **<0.001** |
| DBP (mmHg) | Mean (SD) | 74.6 (11.6) | 64.0 (8.3) | 70.7 (8.2) | 74.9 (8.6) | 78.1 (8.2) | 84.6 (11.5) | **<0.001** |
| Age (years) | Mean (SD) | 65.2 (11.9) | 62.9 (12.8) | 63.1 (12.8) | 65.8 (11.5) | 66.8 (10.5) | 67.5 (10.7) | **<0.001** |
| Female | N (%) | 446 (26.9) | 89 (27.0) | 74 (21.0) | 93 (28.0) | 59 (23.0) | 131 (33.8) | **0.001** |
| Race |  |  |  |  |  |  |  | **0.016** |
| White | N (%) | 1437 (86.6) | 268 (81.2) | 296 (83.9) | 299 (90.1) | 231 (89.9) | 343 (88.4) |  |
| Black | N (%) | 120 (7.2) | 30 (9.1) | 32 (9.1) | 13 (3.9) | 16 (6.2) | 29 (7.5) |  |
| Asian | N (%) | 32 (1.9) | 9 (2.7) | 6 (1.7) | 6 (1.8) | 4 (1.6) | 7 (1.8) |  |
| Other | N (%) | 71 (4.3) | 23 (7.0) | 19 (5.4) | 14 (4.2) | 6 (2.3) | 9 (2.3) |  |
| NYHA functional class |  |  |  |  |  |  |  | **0.005** |
| I | N (%) | 1 (0.1) | 0 (0.0) | 0 (0.0) | 1 (0.3) | 0 (0.0) | 0 (0.0) |  |
| II | N (%) | 5 (0.3) | 1 (0.3) | 0 (0.0) | 3 (0.9) | 0 (0.0) | 1 (0.3) |  |
| III | N (%) | 1171 (70.5) | 205 (62.1) | 249 (70.5) | 233 (70.2) | 187 (72.8) | 297 (76.5) |  |
| IV | N (%) | 483 (29.1) | 124 (37.6) | 104 (29.5) | 95 (28.6) | 70 (27.2) | 90 (23.2) |  |
| Ischemic etiology | N (%) | 906 (54.6) | 168 (50.9) | 199 (56.4) | 185 (55.7) | 141 (54.9) | 213 (55.0) | 0.65 |
| Hypertension | N (%) | 389 (23.4) | 46 (13.9) | 56 (15.9) | 80 (24.1) | 79 (30.7) | 128 (33.0) | **<0.001** |
| Diabetes | N (%) | 368 (22.2) | 51 (15.5) | 69 (19.5) | 59 (17.8) | 76 (29.6) | 113 (29.1) | **<0.001** |
| Previous angina | N (%) | 109 (6.6) | 18 (5.5) | 23 (6.5) | 16 (4.8) | 21 (8.2) | 31 (8.0) | 0.32 |
| Previous MI | N (%) | 472 (28.4) | 92 (27.9) | 103 (29.2) | 97 (29.2) | 77 (30.0) | 103 (26.5) | 0.87 |
| Heart Rate (bpm) | Mean (SD) | 80.9 (14.2) | 81.5 (14.5) | 81.0 (14.7) | 79.9 (14.4) | 81.2 (12.7) | 80.8 (14.2) | 0.67 |
| LVEF (%) | Mean (SD) | 25.4 (6.7) | 23.6 (7.4) | 24.7 (6.8) | 25.6 (6.4) | 26.1 (6.6) | 26.8 (6.0) | **<0.001** |
| Potassium (mg/dL) | Mean (SD) | 4.23 (0.45) | 4.22 (0.42) | 4.20 (0.46) | 4.23 (0.48) | 4.23 (0.41) | 4.25 (0.43) | 0.67 |
| Creatinine (mg/dL) | Mean (SD) | 1.24 (0.36) | 1.27 (0.36) | 1.23 (0.37) | 1.24 (0.36) | 1.23 (0.34) | 1.23 (0.37) | 0.65 |
| eGFR<60 mL/min/1.73m^2^ | N (%) | 789 (47.7) | 167 (50.9) | 143 (40.7) | 169 (51.1) | 121 (47.1) | 189 (48.7) | **0.043** |
| Diuretics | N (%) | 1500 (90.4) | 299 (90.6) | 312 (88.4) | 303 (91.3) | 237 (92.2) | 349 (89.9) | 0.56 |
| ACEi or ARB | N (%) | 1586 (95.5) | 313 (94.8) | 343 (97.2) | 324 (97.6) | 242 (94.2) | 364 (93.8) | **0.046** |
| Betablockers | N (%) | 171 (10.3) | 21 (6.4) | 39 (11.0) | 35 (10.5) | 31 (12.1) | 45 (11.6) | 0.12 |
| Digoxin | N (%) | 1214 (73.1) | 271 (82.1) | 259 (73.4) | 235 (70.8) | 183 (71.2) | 266 (68.6) | **<0.001** |

ACEi denotes angiotensin converting enzyme inhibitor, ARB angiotensin receptor blocker, DBP diastolic blood pressure, eGFR estimated glomerular filtration rate, MI myocardial infarction, NYHA New York heart association class, LVEF left ventricular ejection fraction, SBP systolic blood pressure.

**Table S4. Mean change in SBP from baseline to 1 month and to 6 months, and between-treatment difference in SBP, overall and in each SBP category, in the EMPHASIS-HF trial population.**

|  | Baseline to 1 month | | | Baseline to 6 months | | |
| --- | --- | --- | --- | --- | --- | --- |
| Baseline SBP category | **Placebo** | **MRA** | **Difference** | **Placebo** | **MRA** | **Difference** |
| ≤105 mmHg | 9.9 (13.0) | 6.8 (13.5) | 3.2 | 13.7 (16.7) | 9.9 (15.4) | 4.0 |
| >105 to ≤115 mmHg | 4.5 (12.3) | 3.1 (14.1) | 1.4 | 7.4 (15.8) | 4.7 (15.8) | 2.4 |
| >115 to ≤125 mmHg | 0.0 (12.9) | -0.6 (13.3) | 0.6 | 2.5 (14.8) | 1.7 (14.5) | 0.6 |
| >125 to ≤135 mmHg | -3.5 (13.2) | -4.8 (12.9) | 1.4 | -1.2 (14.3) | -3.7 (13.6) | 2.5 |
| >135 mmHg | -10.0 (15.2) | -12.2 (16.2) | 2.2 | -8.8 (17.8) | -12.0 (17.5) | 3.2 |
| Overall | -0.9 (14.9) | -2.7 (15.6) | 1.8 | 1.4 (17.4) | -1.3 (17.2) | 2.7 |

MRA denotes mineralocorticoid receptor antagonist, SBP systolic blood pressure. Data for each group are reported as mean mmHg change in SBP and standard deviation.

**Table S5. Mean change in SBP from baseline to 1 month and to 6 months, and between-treatment difference in SBP, overall and in each SBP category, in the RALES trial population.**

|  | Baseline to 1 month | | | Baseline to 6 months | | |
| --- | --- | --- | --- | --- | --- | --- |
| Baseline SBP category | **Placebo** | **MRA** | **Difference** | **Placebo** | **MRA** | **Difference** |
| ≤105 mmHg | 7.8 (17.0) | 5.4 (13.4) | 2.4 | 10.4 (15.5) | 9.1 (14.6) | 1.2 |
| >105 to ≤115 mmHg | 3.8 (14.6) | 1.7 (14.9) | 2.1 | 5.3 (17.5) | 4.9 (16.1) | 0.5 |
| >115 to ≤125 mmHg | 2.5 (13.3) | 1.8 (16.7) | 0.7 | 3.7 (15.5) | 3.9 (18.1) | 0.2 |
| >125 to ≤135 mmHg | -2.1 (16.4) | -5.1 (14.4) | 2.9 | 0.0 (20.8) | -3.8 (15.0) | 3.8 |
| >135 mmHg | -10.1 (18.7) | - 11.2 (20.0) | 1.1 | -10.2 (20.1) | -14.7 (18.8) | 4.2 |
| Overall | 0.2 (17.4) | -1.8 (17.4) | 2.1 | 1.3 (19.3) | -1.1 (19.1) | 2.3 |

MRA denotes mineralocorticoid receptor antagonist, SBP systolic blood pressure. Data for each group are reported as mean mmHg change in SBP and standard deviation.

**Table S6. Effect of MRA on clinical outcomes, overall and in each SBP category, in the EMPHASIS-HF trial population.**

|  | Events, n (%) | | | Adjusted hazard ratio  (95% confidence interval), P value | P value for interaction* |
| --- | --- | --- | --- | --- | --- |
| Outcome | **Overall** | **Placebo** | **MRA** |  |  |
| CV death or HF  hospitalization |  |  |  |  | 0.66 |
| ≤105 mmHg | 109 (29.3) | 63 (34.2) | 46 (24.5) | 0.62 (0.41 – 0.94), P=0.023 |  |
| >105 to ≤115 mmHg | 127 (24.6) | 74 (28.5) | 53 (20.6) | 0.59 (0.40 – 0.87), P=0.007 |  |
| >115 to ≤125 mmHg | 146 (23.2) | 92 (28.3) | 54 (17.8) | 0.57 (0.40 – 0.82), P=0.002 |  |
| >125 to ≤135 mmHg | 104 (18.4) | 57 (19.6) | 47 (17.1) | 0.80 (0.53 – 1.20), P=0.28 |  |
| >135 mmHg | 118 (18.1) | 70 (22.4) | 48 (14.1) | 0.63 (0.43 – 0.94), P=0.022 |  |
| Overall | 604 (22.1) | 356 (25.9) | 248 (18.2) | 0.65 (0.55 – 0.77), P<0.001 |  |
| Heart failure  hospitalization |  |  |  |  | 0.90 |
| ≤105 mmHg | 74 (19.9) | 46 (25.0) | 28 (14.9) | 0.55 (0.33 – 0.91), P=0.021 |  |
| >105 to ≤115 mmHg | 100 (19.3) | 60 (23.1) | 40 (15.6) | 0.54 (0.35 – 0.83), P=0.005 |  |
| >115 to ≤125 mmHg | 99 (15.8) | 64 (19.7) | 35 (11.6) | 0.56 (0.36 – 0.87), P=0.010 |  |
| >125 to ≤135 mmHg | 65 (11.5) | 37 (12.7) | 28 (10.2) | 0.71 (0.42 – 1.22), P=0.22 |  |
| >135 mmHg | 79 (12.1) | 46 (14.7) | 33 (9.7) | 0.64 (0.39 – 1.04), P=0.071 |  |
| Overall | 417 (15.2) | 253 (18.4) | 164 (12.0) | 0.61 (0.50 – 0.74), P<0.001 |  |
| Cardiovascular death |  |  |  |  | 0.91 |
| ≤105 mmHg | 68 (18.3) | 36 (19.6) | 32 (17.0) | 0.77 (0.45 – 1.30), P=0.32 |  |
| >105 to ≤115 mmHg | 65 (12.6) | 38 (14.6) | 27 (10.5) | 0.69 (0.40 – 1.18), P=0.18 |  |
| >115 to ≤125 mmHg | 81 (12.9) | 46 (14.2) | 35 (11.6) | 0.78 (0.49 – 1.26), P=0.31 |  |
| >125 to ≤135 mmHg | 61 (10.8) | 32 (11.0) | 29 (10.5) | 0.79 (0.46 – 1.36), P=0.40 |  |
| >135 mmHg | 56 (8.6) | 33 (10.5) | 23 (6.8) | 0.67 (0.38 – 1.17), P=0.16 |  |
| Overall | 331 (12.1) | 185 (13.5) | 146 (10.7) | 0.75 (0.60 – 0.94), P=0.011 |  |
| All-cause death |  |  |  |  | 0.86 |
| ≤105 mmHg | 79 (21.2) | 45 (24.5) | 34 (18.1) | 0.63 (0.38 – 1.02), P=0.061 |  |
| >105 to ≤115 mmHg | 78 (15.1) | 44 (16.9) | 34 (13.2) | 0.76 (0.47 – 1.24), P=0.271 |  |
| >115 to ≤125 mmHg | 96 (15.3) | 53 (16.3) | 43 (14.2) | 0.83 (0.54 – 1.28), P=0.405 |  |
| >125 to ≤135 mmHg | 67 (11.8) | 36 (12.4) | 31 (11.3) | 0.74 (0.44 – 1.23), P=0.244 |  |
| >135 mmHg | 63 (9.6) | 35 (11.2) | 28 (8.2) | 0.81 (0.48 – 1.36), P=0.422 |  |
| Overall | 383 (14.0) | 213 (15.5) | 170 (12.5) | 0.76 (0.61 – 0.93), P=0.009 |  |

CV death denotes cardiovascular death, HF hospitalization heart failure hospitalization, MRA mineralocorticoid receptor antagonist, SBP systolic blood pressure.

*P value is for interaction between SBP categories and treatment effect.

**Table S7. Effect of MRA on clinical outcomes, overall and in each SBP category, in the RALES trial population.**

|  | Events, n (%) | | | Adjusted hazard ratio  (95% confidence interval), P value | P value for interaction* |
| --- | --- | --- | --- | --- | --- |
| Outcome | **Overall** | **Placebo** | **MRA** |  |  |
| CV death or HF  hospitalization |  |  |  |  | 0.063 |
| ≤105 mmHg | 203 (61.5) | 113 (63.1) | 90 (59.6) | 0.81 (0.61 – 1.08), P=0.16 |  |
| >105 to ≤115 mmHg | 192 (54.4) | 103 (60.2) | 89 (48.9) | 0.82 (0.60 – 1.11), P=0.19 |  |
| >115 to ≤125 mmHg | 147 (44.3) | 85 (50.0) | 62 (38.3) | 0.68 (0.48 – 0.96), P=0.03 |  |
| >125 to ≤135 mmHg | 118 (45.9) | 72 (55.0) | 46 (36.5) | 0.60 (0.40 – 0.88), P=0.01 |  |
| >135 mmHg | 157 (40.5) | 99 (52.1) | 58 (29.3) | 0.43 (0.31 – 0.61), P<0.001 |  |
| Overall | 817 (49.2) | 472 (56.1) | 345 (42.1) | 0.66 (0.57 – 0.76), P<0.001 |  |
| Heart failure  hospitalization |  |  |  |  | 0.056 |
| ≤105 mmHg | 118 (35.8) | 67 (37.4) | 51 (33.8) | 0.73 (0.49 – 1.07), P=0.10 |  |
| >105 to ≤115 mmHg | 127 (36.0) | 71 (41.5) | 56 (30.8) | 0.71 (0.49 – 1.03), P=0.067 |  |
| >115 to ≤125 mmHg | 90 (27.1) | 46 (27.1) | 44 (27.2) | 0.85 (0.55 – 1.31), P=0.47 |  |
| >125 to ≤135 mmHg | 74 (28.8) | 47 (35.9) | 27 (21.4) | 0.51 (0.31 – 0.85), P=0.009 |  |
| >135 mmHg | 106 (27.3) | 69 (36.3) | 37 (18.7) | 0.40 (0.26 – 0.60), P<0.001 |  |
| Overall | 515 (31.0) | 300 (35.7) | 215 (26.3) | 0.65 (0.54 – 0.78), P<0.001 |  |
| Cardiovascular death |  |  |  |  | 0.54 |
| ≤105 mmHg | 148 (44.8) | 83 (46.4) | 65 (43.0) | 0.80 (0.57 – 1.12), P=0.18 |  |
| >105 to ≤115 mmHg | 135 (38.2) | 72 (42.1) | 63 (34.6) | 0.95 (0.66 – 1.37), P=0.79 |  |
| >115 to ≤125 mmHg | 97 (29.2) | 61 (35.9) | 36 (22.2) | 0.61 (0.40 – 0.95), P=0.027 |  |
| >125 to ≤135 mmHg | 70 (27.2) | 44 (33.6) | 26 (20.6) | 0.61 (0.37 – 1.02), P=0.060 |  |
| >135 mmHg | 89 (22.9) | 54 (28.4) | 35 (17.7) | 0.58 (0.37 – 0.90), P=0.015 |  |
| Overall | 539 (32.5) | 314 (37.3) | 225 (27.5) | 0.69 (0.58 – 0.82), P<0.001 |  |
| All-cause death |  |  |  |  | 0.85 |
| ≤105 mmHg | 174 (52.7) | 99 (55.3) | 75 (49.7) | 0.77 (0.57 – 1.06), P=0.11 |  |
| >105 to ≤115 mmHg | 165 (46.7) | 91 (53.2) | 74 (40.7) | 0.84 (0.61 – 1.17), P=0.31 |  |
| >115 to ≤125 mmHg | 118 (35.5) | 72 (42.4) | 46 (28.4) | 0.64 (0.43 – 0.95), P=0.025 |  |
| >125 to ≤135 mmHg | 87 (33.9) | 50 (38.2) | 37 (29.4) | 0.79 (0.50 – 1.23), P=0.29 |  |
| >135 mmHg | 125 (32.2) | 74 (38.9) | 51 (25.8) | 0.61 (0.43 – 0.89), P=0.009 |  |
| Overall | 669 (40.3) | 386 (45.9) | 283 (34.6) | 0.70 (0.60 – 0.82), P<0.001 |  |

CV Death denotes cardiovascular death, MRA mineralocorticoid receptor antagonist, SBP systolic blood pressure. P value is for interaction between SBP categories and treatment effect.

**Table S8. Rate of adverse effects and permanent study drug discontinuation according to treatment in the EMPHASIS-HF trial population.**

|  | Adverse Effects, n (%) | | |  |
| --- | --- | --- | --- | --- |
| Variable | **Overall**  **(N=2736)** | **Placebo**  **(N=1373)** | **MRA**  **(N=1363)** | **P value** |
| Hypotension | 101 (3.7) | 47 (3.4) | 54 (4.0) | 0.45 |
| SBP drop ≥30 mmHg to 6 months | 183 (6.7) | 79 (5.8) | 104 (7.6) | **0.05** |
| SBP <85 mmHg* at 1 month | 14 (0.5) | 6 (0.4) | 8 (0.6) | 0.59 |
| SBP <85 mmHg* at 6 months | 14 (0.5) | 2 (0.2) | 12 (0.9) | **0.007** |
| SBP <85 mmHg* at 1 month and 6 months | 3 (0.1) | 0 (0.0) | 3 (0.2) | 0.082 |
| Creatinine ≥ 2.5 mg/dL | 50 (1.8) | 22 (1.6) | 28 (2.1) | 0.38 |
| Creatinine ≥ 3.0 mg/dL | 20 (0.7) | 11 (0.8) | 9 (0.7) | 0.67 |
| Potassium > 5.5 mmol/L | 254 (9.3) | 94 (6.8) | 160 (11.7) | **<0.001** |
| Potassium > 6.0 mmol/L | 57 (2.1) | 23 (1.7) | 34 (2.5) | 0.13 |
| Discontinuation of study drug | 424 (15.5) | 225 (16.4) | 199 (14.6) | 0.20 |

MRA denotes mineralocorticoid receptor antagonist, SBP systolic blood pressure.

*Patients with baseline SBP lower than 85 mmHg were excluded from this analysis

**Table S9. Rate of adverse effects and permanent study drug discontinuation according to treatment in the RALES trial population.**

|  | Adverse Effects, n (%) | | | |
| --- | --- | --- | --- | --- |
| Variable | **Overall**  **(N=1663)** | **Placebo**  **(N=841)** | **MRA**  **(N=822)** | **P value** |
| Hypotension | 85 (5.1) | 39 (4.6) | 46 (5.6) | 0.37 |
| SBP drop ≥30 mmHg to 6 months | 156 (9.4) | 71 (8.4) | 85 (10.4) | 0.18 |
| SBP <85 mmHg* at 1 month | 23 (1.4) | 10 (1.2) | 13 (1.6) | 0.49 |
| SBP <85 mmHg* at 6 months | 15 (0.9) | 10 (1.2) | 5 (0.6) | 0.21 |
| SBP <85 mmHg* at 1 month and 6 months | 2 (0.1) | 2 (0.2) | 0 (0.0) | 0.16 |
| Creatinine ≥ 2.5 mg/dL | 113 (6.8) | 43 (5.1) | 70 (8.5) | **0.005** |
| Creatinine ≥ 3.0 mg/dL | 47 (2.8) | 17 (2.0) | 30 (3.7) | **0.044** |
| Potassium > 5.5 mmol/L | 165 (9.9) | 38 (4.5) | 127 (15.5) | **<0.001** |
| Potassium > 6.0 mmol/L | 41 (2.5) | 9 (1.1) | 32 (3.9) | **<0.001** |
| Discontinuation study drug | 452 (27.2) | 216 (25.7) | 236 (28.8) | 0.15 |

MRA denotes mineralocorticoid receptor antagonist, SBP systolic blood pressure.

*Patients with baseline SBP lower than 85 mmHg were excluded from this analysis

**Table S10.** **Rate of adverse effects and permanent study drug discontinuation according treatment and SBP categories in the EMPHASIS-HF trial population.**

|  | Adverse Effects, n (%) | | | | | | | | | |  |
| --- | --- | --- | --- | --- | --- | --- | --- | --- | --- | --- | --- |
| Variable | **≤105 mmHg** | | **>105 to ≤115 mmHg** | | **>115 to ≤125 mmHg** | | **>125 to ≤135 mmHg** | | **>135 mmHg** | | **P**  **value** |
|  | **Placebo**  **(N=184)** | **MRA**  **(N=188)** | **Placebo**  **(N=260)** | **MRA**  **(N=257)** | **Placebo**  **(N=325)** | **MRA**  **(N=303)** | **Placebo**  **(N=291)** | **MRA**  **(N=275)** | **Placebo**  **(N=313)** | **MRA**  **(N=340)** |  |
| Hypotension | 16 (8.7) | 19 (10.1) | 10 (3.8) | 8 (3.1) | 9 (2.8) | 12 (4.0) | 7 (2.4) | 5 (1.8) | 5 (1.6) | 10 (2.9) | 0.71 |
| SBP drop ≥30 mmHg to 6 months | 0 (0.0) | 1 (0.5) | 1 (0.4) | 3 (1.2) | 2 (0.6) | 11 (3.6) | 15 (5.2) | 16 (5.8) | 61 (10.5) | 73 (21.5) | -^†^ |
| SBP <85 mmHg* at 1 month | 4 (2.2) | 5 (2.7) | 1 (0.4) | 3 (1.2) | 0 (0.0) | 0 (0.0) | 1 (0.3) | 0 (0.0) | 0 (0.0) | 0 (0.0) | -^†^ |
| SBP <85 mmHg* at 6 months | 2 (1.1) | 9 (4.8) | 0 (0.0) | 2 (0.8) | 0 (0.0) | 0 (0.0) | 0 (0.0) | 0 (0.0) | 0 (0.0) | 1 (0.3) | -^†^ |
| SBP <85 mmHg* at 1 month and 6 months | 0 (0.0) | 2 (1.1) | 0 (0.0) | 1 (0.4) | 0 (0.0) | 0 (0.0) | 0 (0.0) | 0 (0.0) | 0 (0.0) | 0 (0.0) | -^†^ |
| Creatinine ≥ 2.5 mg/dL | 4 (2.2) | 7 (3.7) | 4 (1.5) | 6 (2.3) | 8 (2.5) | 5 (1.7) | 1 (0.3) | 3 (1.1) | 5 (1.6) | 7 (2.1) | 0.70 |
| Creatinine ≥ 3.0 mg/dL | 2 (1.1) | 3 (1.6) | 1 (0.4) | 1 (0.4) | 3 (0.9) | 2 (0.7) | 1 (0.3) | 1 (0.4) | 4 (1.3) | 2 (0.6) | 0.91 |
| Potassium > 5.5 mmol/L | 12 (6.5) | 21 (11.2) | 18 (6.9) | 21 (8.2) | 19 (5.8) | 39 (12.9) | 21 (7.2) | 38 (13.8) | 24 (7.7) | 41 (12.1) | 0.60 |
| Potassium > 6.0 mmol/L | 4 (2.2) | 4 (2.1) | 5 (1.9) | 4 (1.6) | 5 (1.5) | 9 (3.0) | 3 (1.0) | 7 (2.5) | 6 (1.9) | 10 (2.9) | 0.73 |
| Discontinuation of study drug | 40 (21.7) | 25 (13.3) | 58 (22.3) | 52 (20.2) | 48 (14.8) | 43 (14.2) | 36 (12.4) | 28 (10.2) | 43 (13.7) | 51 (15.0) | 0.39 |

MRA denotes mineralocorticoid receptor antagonist, SBP systolic blood pressure. P-values are for interaction between systolic blood pressure categories and treatment effect.

*Patients with baseline SBP lower than 85 mmHg were excluded from this analysis

†P value not provided because of few events

**Table S11.** **Rate of adverse effects and permanent study drug discontinuation according treatment and SBP categories in the RALES trial population.**

|  | Adverse Effects, n (%) | | | | | | | | | |  |
| --- | --- | --- | --- | --- | --- | --- | --- | --- | --- | --- | --- |
| Variable | **≤105 mmHg** | | **>105 to ≤115 mmHg** | | **>115 to ≤125 mmHg** | | **>125 to ≤135 mmHg** | | **>135 mmHg** | | **P value** |
|  | **Placebo**  **(N=179)** | **MRA**  **(N=151)** | **Placebo**  **(N=171)** | **MRA**  **(N=182)** | **Placebo**  **(N=170)** | **MRA**  **(N=162)** | **Placebo**  **(N=131)** | **MRA**  **(N=126)** | **Placebo**  **(N=190)** | **MRA**  **(N=198)** |  |
| Hypotension | 16 (8.9) | 16 (10.6) | 7 (4.1) | 10 (5.5) | 7 (4.1) | 10 (6.2) | 6 (4.6) | 4 (3.2) | 3 (1.6) | 6 (3.0) | 0.83 |
| SBP drop ≥30 mmHg to 6 months | 0 (0.0) | 0 (0.0) | 2 (1.2) | 2 (1.1) | 3 (1.8) | 7 (4.3) | 12 (9.2) | 12 (9.5) | 54 (28.4) | 64 (32.3) | 0.72 |
| SBP <85 mmHg* at 1 month | 10 (5.8) | 8 (5.6) | 0 (0.0) | 2 (1.1) | 0 (0.0) | 2 (1.2) | 0 (0.0) | 1 (0.8) | 0 (0.0) | 0 (0.0) | -^†^ |
| SBP <85 mmHg* at 6 months | 7 (4.1) | 5 (3.5) | 2 (1.2) | 0 (0.0) | 0 (0.0) | 0 (0.0) | 0 (0.0) | 0 (0.0) | 1 (0.5) | 0 (0.0) | -^†^ |
| SBP <85 mmHg* at 1 month and 6 months | 2 (1.2) | 0 (0.0) | 0 (0.0) | 0 (0.0) | 0 (0.0) | 0 (0.0) | 0 (0.0) | 0 (0.0) | 0 (0.0) | 0 (0.0) | -^†^ |
| Creatinine ≥ 2.5 mg/dL | 9 (5.0) | 19 (12.6) | 11 (6.4) | 13 (7.1) | 7 (4.1) | 12 (7.4) | 6 (4.6) | 8 (6.3) | 10 (5.3) | 18 (9.1) | 0.66 |
| Creatinine ≥ 3.0 mg/dL | 4 (2.2) | 12 (7.9) | 5 (2.9) | 3 (1.6) | 3 (1.8) | 2 (1.2) | 1 (0.8) | 4 (3.2) | 4 (2.1) | 9 (4.5) | 0.18 |
| Potassium > 5.5 mmol/L | 3 (1.7) | 15 (9.9) | 9 (5.3) | 27 (14.8) | 9 (5.3) | 28 (17.3) | 3 (2.3) | 19 (15.1) | 14 (7.4) | 38 (19.2) | 0.58 |
| Potassium > 6.0 mmol/L | 0 (0.0) | 6 (4.0) | 3 (1.8) | 7 (3.8) | 2 (1.2) | 2 (1.2) | 0 (0.0) | 3 (2.4) | 4 (2.1) | 14 (7.1) | -^†^ |
| Withdrawal from study drug | 45 (25.1) | 47 (31.1) | 38 (22.2) | 46 (25.3) | 38 (22.4) | 45 (27.8) | 46 (35.1) | 36 (28.6) | 49 (25.8) | 62 (31.3) | 0.44 |

MRA denotes mineralocorticoid receptor antagonist, SBP systolic blood pressure. P-values are for interaction between systolic blood pressure categories and treatment effect.

*Patients with baseline SBP lower than 85 mmHg were excluded from this analysis

†P value not provided because of few events

**Figure S1. Hazard ratio for (A) Cardiovascular Death or First Hospitalization for Heart Failure, (B) Hospitalization for Heart Failure, (C) Cardiovascular Death and (D) All-Cause Death in patient treated with MRA, according to baseline SBP.**


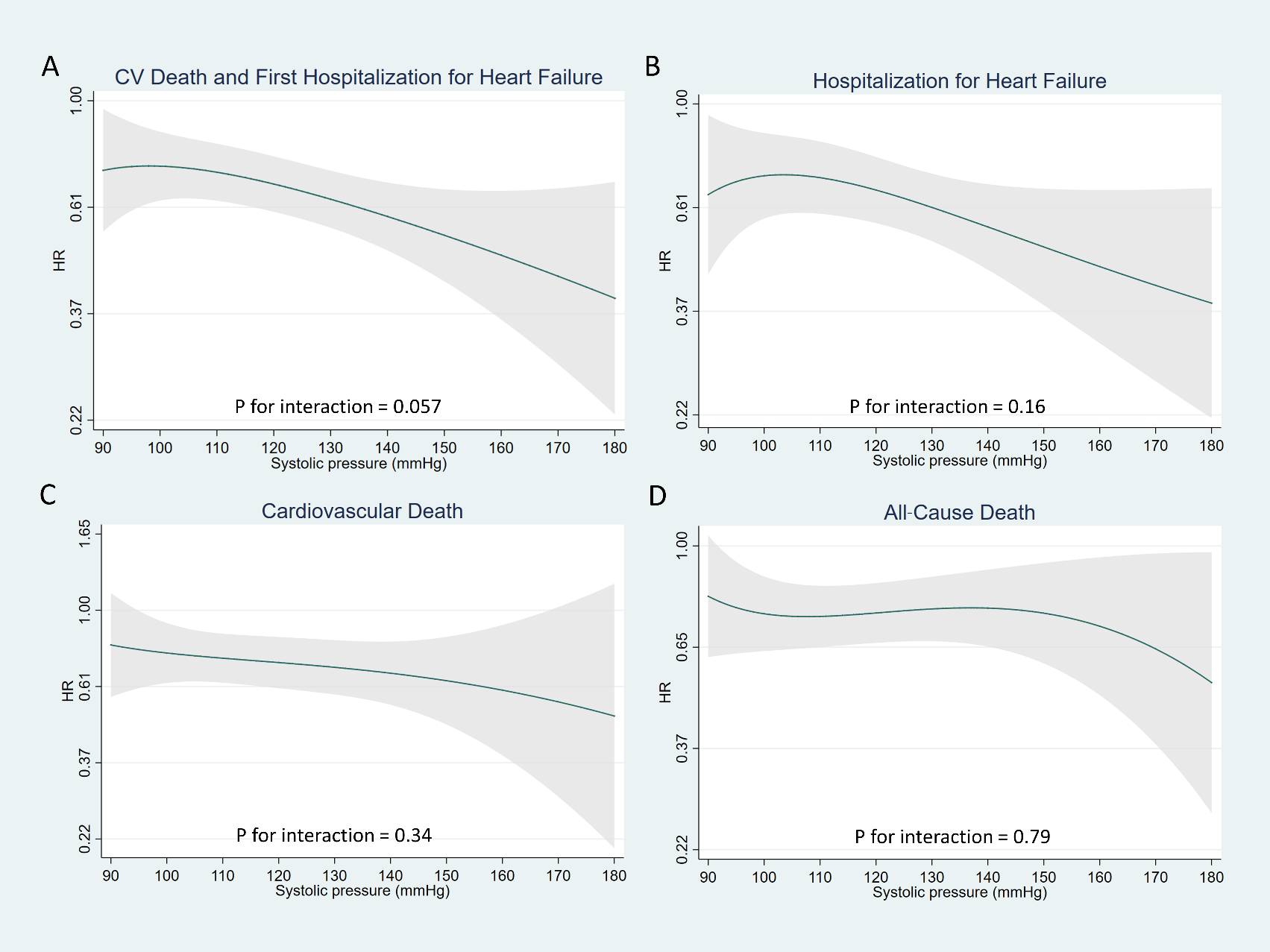


*P-values are for interaction between systolic blood pressure and treatment effect.

**Figure S2. Hazard ratio for (A) Cardiovascular Death or First Hospitalization for Heart Failure, (B) Hospitalization for Heart Failure, (C) Cardiovascular Death and (D) All-Cause Death in patient treated with MRA, according to baseline SBP in the EMPHASIS-HF trial population.**


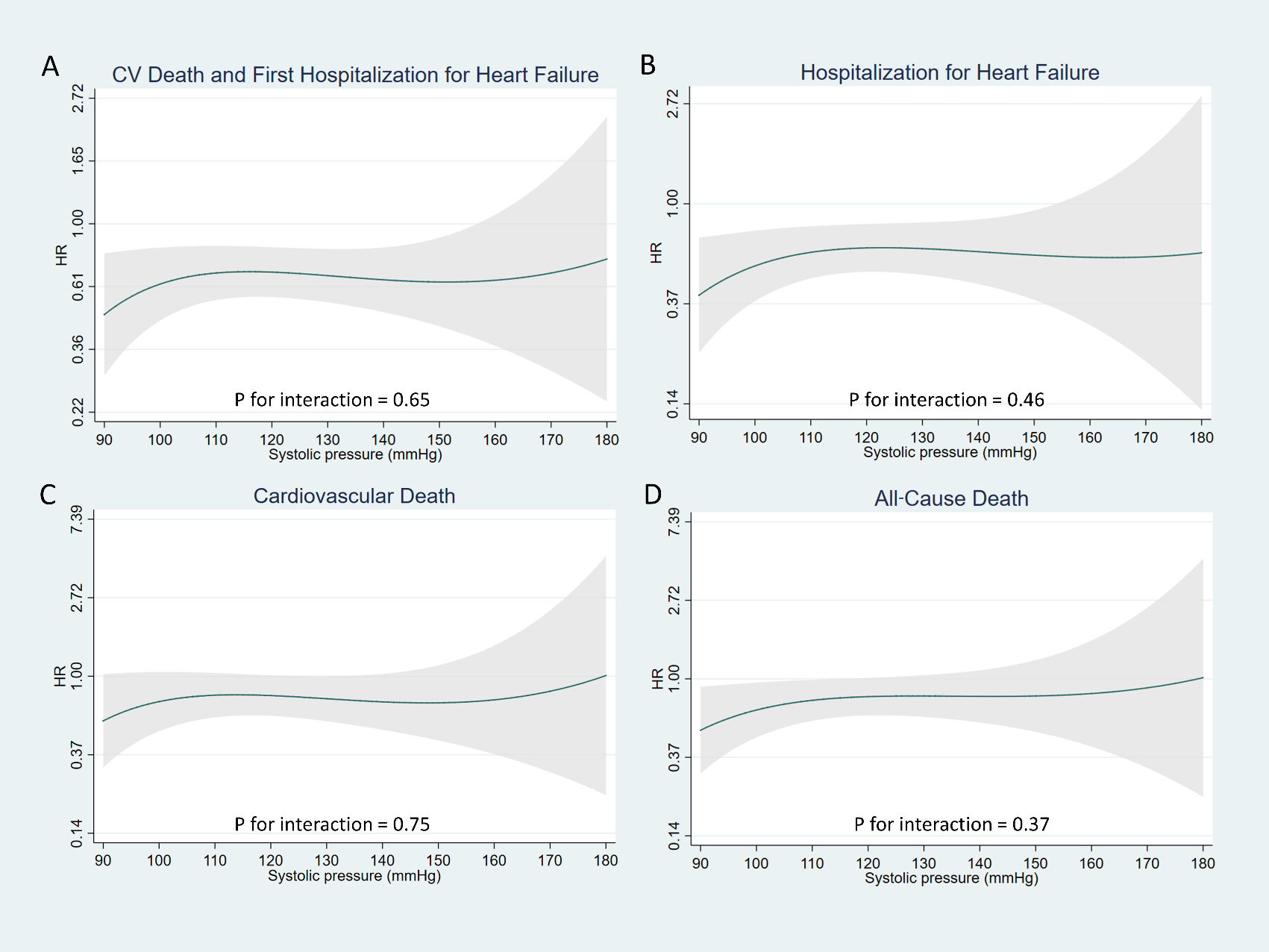


*P-values are for interaction between systolic blood pressure and treatment effect.

**Figure S3. Hazard ratio for (A) Cardiovascular Death or First Hospitalization for Heart Failure, (B) Hospitalization for Heart Failure, (C) Cardiovascular Death and (D) All-Cause Death in patient treated with MRA, according to baseline SBP in the RALES trial population.**


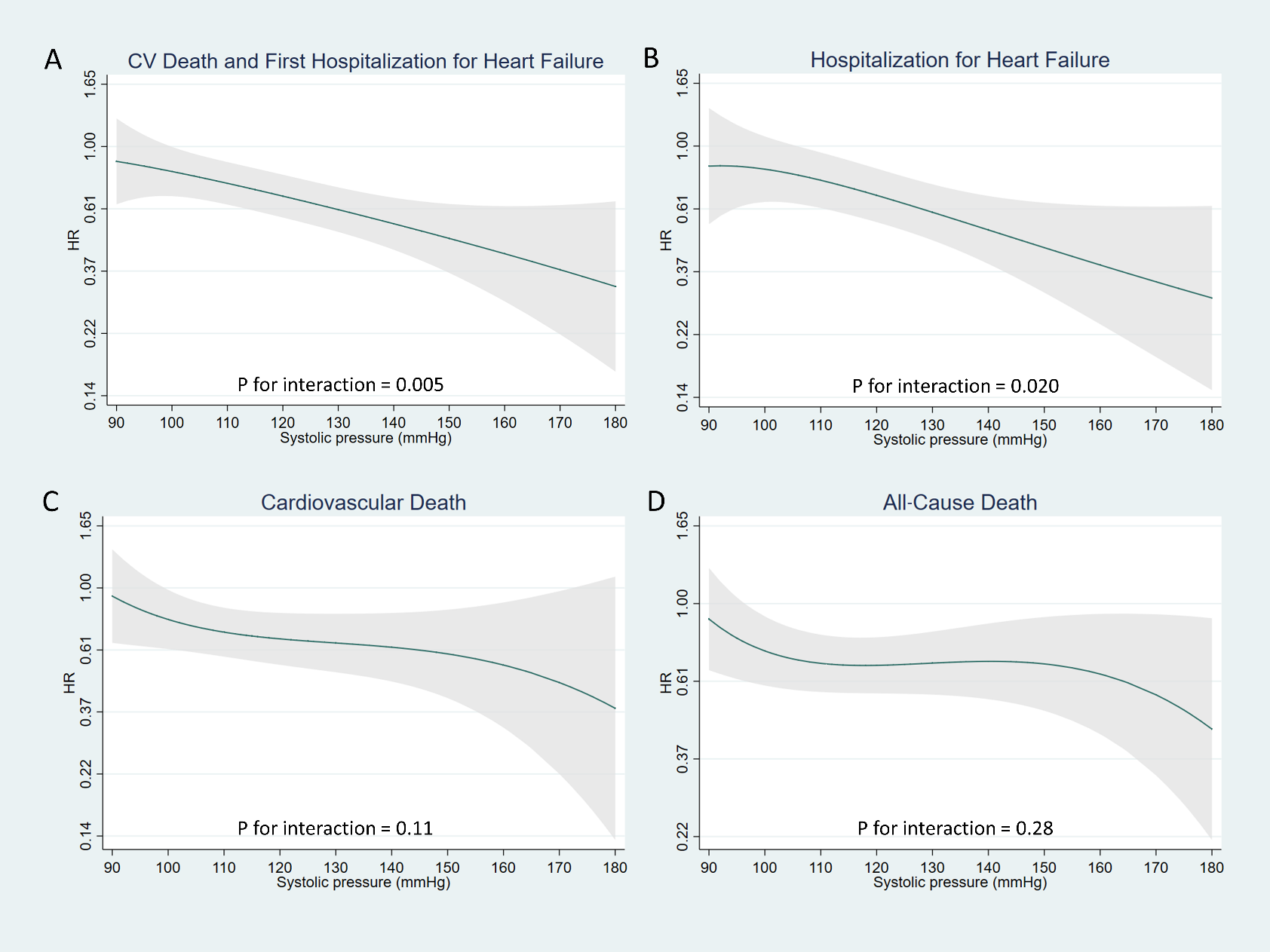


*P-values are for interaction between systolic blood pressure and treatment effect.
